# Commentary: Cultural differences in on-line sensitivity to emotional voices: comparing East and West

**DOI:** 10.3389/fnhum.2015.00696

**Published:** 2016-01-19

**Authors:** István Czigler

**Affiliations:** Centre for Natural Sciences, Institute of Cognitive Neuroscience and Psychology, Hungarian Academy of SciencesBudapest, Hungary

**Keywords:** facial emotions, cultural differences, visual mismatch negativity, automatic detection, speech sensitivity

On the basis of their visual mismatch negativity (vMMN) results, Liu et al. (2015) claim that processing of facial emotions is different between Chinese (and more generally “Eastern”) and Canadian (and more generally “Western”) people. Simply put, Eastern people are more sensitive to non-visual aspects of the environment, e.g., to the emotional tone of speech presented in the background of photographs of faces. I do not want to deny the possibility of such difference. However, I feel that the data by Liu et al. (2015) do not substantiate their claim.

In short, they applied a modified three-stimulus oddball task, with circles as target stimuli and faces as non-target stimuli. There were photographs among the faces showing a frequent emotion category (standard) and a rare one (deviant). Such sequences were presented to Chinese and Canadian participants; same race photographs were used for each group. In the first condition, only visual stimuli were presented; in the second, the photographs were accompanied by meaningless emotional speech (congruent or incongruent with the photographs); and in the third, the auditory stimuli were tones. As for the details of stimulus presentation, EEG recording, etc., this study corresponds to the professional standard. The problematic issues are (1) the relationship between the present findings and the specific requirements of vMMN research; (2) the connection between the data and the interpretation.

1. VMMN is considered as an index of an automatic process, elicited by the violation of regular stimulation. This is why in vMMN studies a primary task is introduced in order to distract attention from the vMMN-related stimuli (see Czigler, 2007 for a discussion). In the Liu et al. (2015) study single faces were presented in the center of an otherwise empty field for 800 ms with 650 ms mean ISI. Is it possible “not to attend” (ignore) such photographs? Are there any sophisticated adult participants who do not suspect that such salient events are an important part of the study? Even if the sequence of photographs becomes a bit boring, simultaneous presentations of the speech-like stimuli are supposed to exert alerting effects. As a conclusion, any deviant effects in this paradigm seem to be driven by a mixture of automatic and attentional processing. What I suggest for further research in this field is the application of more stringent control of attention. In some studies (Li et al., 2012; Stefanics et al., 2012) the task was presented in the center of the visual field, and the faces appeared beside the task field or at the edges of an imagery square. In case of central presentation of the vMMN-related stimuli, a continuous task with stimuli independent of the appearance the faces (e.g., Kecskes-Kovacs et al., 2013) seems to be appropriate for diverting attention. It should be noted that control of attention and the attentional effects on MMN were important issues the auditory MMN, even if diverting attention from auditory stimuli with visual events (silent movies, reading of interesting books) is fairly successful (for a discussion of the attention issue in the auditory modality from theoretical point of view see Sussman et al., 2014; and for the technical aspects see Campbell, 2015).
2. The main results of the Liu et al. (2015) study are shown on their Figure 4. As the records in this figure indicate, in the 100–200 ms range in the speech-like condition the voltage maximum of the Chinese participants increased. The interpretation of this result deserves some comments.

In case of a baseline-peak measure, there is no problem with these results. However, as this figure shows, the onset on the difference potentials was much earlier and in fact differences seemed to appear even before stimulus onset. The origin of the early effect is unaccounted for and it produces an offset, which casts a shadow on the interpretation of the subsequent differential effect as being a modulation of the MMN. Furthermore, it is obvious that in the Chinese group the negative shift observed is long lasting, rather than a modulation circumscribed to a particular range. As Figure 4 shows, the difference potentials were just as different in later latency ranges as within the 100–200 ms window. In both groups the negativity was longer in the speech-related condition, and with the tones it seems to be larger in the Canadian sample. Unfortunately, the authors did not report or discuss the later effects.

In the language-related condition there were two types of sequences. In one of the sequences the speech-related and the face-related emotions were congruent, and in the other condition they were incongruent. Presumably (and reasonably) congruency was considered to be an important factor (as it was entered into the ANOVAs) but this factor had no effects on the ERPs. Thus, emotional content did not help Chinese participants to identify emotional contents, which directly contradicts the main hypothesis of the study.

Despite measurable group differences in this study, and even though “Eastern-Western” differences most likely exist in the perception of facial emotions, the ERP effect reported cannot be unequivocally interpreted as culture-related differences in the processing of facial expressions. In a convincing study, as the first step, it is important to disclose how the two groups perceived the experimental situation (in this particular case the presence of faces and the additional presence of speech). As for the source of differences numerous *ad hoc* hypotheses can be constructed. For example, participants might search for the meaning of the utterances, and this strategy may have been different in the two samples; utterances in the two languages may have had different arousing capacity, etc.

It remains that mismatch-related ERP effects (both in the auditory and in the visual modality) are exceptional tools in the investigation of automatic processing. Furthermore, learned processing strategies probably influence the acquisition of the memory system underlying mismatch responses, as has been shown in several studies (e.g., Thierry et al., 2009; Mo et al., 2011). On the basis of such sensitivity, it is possible to construct experiments which address broad questions beyond the methodological realm of “mismatch specialists.” In my opinion, special care should be taken when designing and analyzing data which have been collected to address questions with broad impact.

## Funding

Supported by the Hungarian Research Fund (OTKA) 104462.

### Conflict of interest statement

The author declares that the research was conducted in the absence of any commercial or financial relationships that could be construed as a potential conflict of interest.
